# Long-Term Trends in Stroke Management and Burden Among Low-Income Women in a Rural Area From China (1992–2019): A Prospective Population-Based Study

**DOI:** 10.3389/fneur.2021.720962

**Published:** 2021-10-22

**Authors:** Xiaobing Tian, Jie Liu, Changshen Yu, Yabing Hou, Changqing Zhan, Qiuxing Lin, Xinyu Zhang, Xin Zhang, Dandan Guo, Qiaoxia Yang, Jun Tu, Yaogang Wang, Xianjia Ning, Jinghua Wang

**Affiliations:** ^1^Department of Neurology, Tianjin Medical University General Hospital, Tianjin, China; ^2^Laboratory of Epidemiology, Tianjin Neurological Institute, Tianjin, China; ^3^Key Laboratory of Post-neuroinjury Neuro-Repair and Regeneration in Central Nervous System, Tianjin Neurological Institute, Ministry of Education and Tianjin City, Tianjin, China; ^4^Department of Neurology, Tianjin Huanhu Hospital, Tianjin, China; ^5^School of Public Health, Tianjin Medical University, Tianjin, China; ^6^Department of Neurology, The Second People's Hospital of Wuhu, Wuhu, China; ^7^Department of Cardiology, Tianjin Medical University General Hospital, Tianjin, China; ^8^Public Health Science and Engineering College, Tianjin University of Traditional Chinese Medicine, Tianjin, China

**Keywords:** stroke, burden, management, trends, women

## Abstract

Although an increasing number of studies are considering sex-related differences in stroke burden, the trends in stroke burden and management among women in China, especially among low-income women, remain unclear. This study evaluated the long-term trends in stroke management and burden among low-income Chinese women during the period between 1992 and 2019. Stroke burden was assessed using the age-adjusted incidence of first-ever stroke, whereas stroke management was assessed using the rates of neuroimaging diagnoses, hospitalizations, case fatalities, and stroke recurrence. Stroke burden and management were analyzed during four study periods: 1992–1998, 1999–2004, 2005–2012, and 2013–2019. During the 193,385 person-years of surveillance in this study, 597 female stroke patients were identified. The stroke incidences per 100,000 person-years were 88.1 cases during 1992–1998, 145.4 cases during 1999–2004, 264.3 cases during 2005–2012, and 309.8 cases during 2013–2019 (*P* < 0.001). Between 1992 and 2019, the incidence of stroke significantly increased (6.4% annually) as did the incidence of ischemic stroke (7.8% annually; both, *P* < 0.001). The rates of neuroimaging diagnoses and hospitalizations significantly increased during the four periods, while the case fatality rates and 1-year recurrence rates decreased significantly for both overall strokes and ischemic strokes, especially among patients ≥45 years old (all, *P* < 0.001). Among low-income women in China, stroke management is gradually improving, despite the increasing stroke burden. Thus, improved healthcare coverage is needed to further reduce the stroke burden among low-income Chinese women.

## Introduction

After cardiovascular disease, stroke is the second leading cause of death and lost disability-adjusted life-years (DALYs), worldwide (1, 2). This burden is particularly prominent in low-income countries, which account for 78% of DALYs lost to stroke, and this rate is at least 7-fold higher than the DALYs lost in high-income countries (3). China is a developing country that has experienced rapid increases in the incidence and burden of stroke, with stroke-associated deaths becoming the leading cause of mortality in China (4). Thus, stroke creates a heavy burden on Chinese society, families, and especially women; China's National Bureau of Statistics data indicate that cerebrovascular disease remains the second leading cause of death among women in rural China (5).

Although there was a sharp, global reduction in stroke-related deaths between 1990 and 2016 (1), the long-term effects of stroke are still very concerning, especially for women. Among individuals 55–75 years old, these effects are significantly greater for men than for women, although the prevalence of stroke among women is rapidly increasing and reached 41 million in 2016 (vs. 39 million among men) (1). Results from the Framingham cohort also revealed that women have a higher lifetime risk of stroke than men (6), while a large American cohort study revealed that the incidences of stroke and ischemic stroke (IS) decrease over time among men but not among women (7). Moreover, women experience greater disability after stroke (8) and the 2014 Guidelines for the Prevention of Stroke in Women suggest that these sex-based differences may be related to women having unique vascular profiles and risk factors (vs. men) that are related to immunity (9, 10), coagulation (11, 12), reproductive factors (13), and social factors (14). Although an increasing number of studies have examined the sex-based differences in stroke risk and burden (7, 8, 13, 15–18), most studies have focused on high-income countries and few have considered low-income areas (19). In addition, we are not aware of any studies regarding the stroke-burden trends among rural Chinese women. Therefore, this study evaluated data from a 28-year stroke surveillance project to determine the long-term trends in stroke management and burden among low-income women in China during the 1992–2019 period.

## Methods

### Study Population

This population-based study evaluated data from the Tianjin Brain Study (TBS), which is an ongoing population-based stroke surveillance study conducted in a township of Ji county (Tianjin, China). The study population is all the permanent residents of 18 administrative villages in Jizhou District, Tianjin, China. Through the local three-level disease reporting system (village doctor-health center-hospital), the incidence of stroke patients and the prognosis in the later period are recorded and uploaded in a timely manner. In this study, we analyzed the long-term prognosis, imaging diagnosis rate, and hospitalization rate of this population from 1992 to 2019. Despite the TBS being launched in 1985, we excluded data from 1985 to 1991 because computed tomography (CT) was only introduced to this region in 1992. The study's detailed methodology has been previously reported (20–23). Briefly, the study included all residents from 18 administrative villages in Tianjin township. In the study area, 95% of residents were low-income farmers (grain production) (5) with annual per capita incomes of < $100 US dollars (USD) in 1991 and < $2500 USD in 2018. Population information was obtained from the Center for Disease Control and Prevention in Jizhou District, Tianjin. The total female population of the region was 7,169 (accounted for 48.0% of all population) in 1992 and 6,815 (accounted for 46.8% of all population) in 2019, respectively. The local illiteracy rate in women aged ≥45 years is as high as 23.4% (24). Moreover, after the 2008 national medical reform in this region, the medical insurance coverage rate increased from 20% (2008) to 100% (2009) (25).

The study protocol was approved by the ethics committee of Tianjin Medical University General Hospital (TMUGH). All subjects provided written informed consent, or consent was obtained from an appropriate family member in situations where the patient was disabled and unable to consent.

### Medical Insurance Information

There are two types of medical insurance in China: the formal sector employee medical insurance scheme and the resident medical insurance scheme. Tianjin's urban and rural residents' medical insurance reached universal coverage after the 2009 health care reform; (26, 27) before that, medical insurance was scarce in rural areas. Since 2008, the resident medical insurance was available to the study population.

### Data Collection

Annual data were collected regarding demographic characteristics; medical histories of hypertension, diabetes, and disability (excluding cases involving death, missing data, or a modified Rankin Scale score of >2) (28); and rates of neuroimaging diagnoses and hospitalizations. All symptomatic stroke events and all-cause deaths were registered in real time. Data were predominantly acquired via face-to-face interviews with patients or their relatives, and were collected by senior neurologists.

### Identifying Stroke Events

Predefined procedures were used to identify stroke events, which included first-ever stroke and stroke recurrence (23). Local village physicians initially reported possible stroke events to the community hospital within 24 h after stroke onset. The community hospital physicians subsequently visited the patients' homes to obtain clinical information and perform initial diagnoses, which included recording histories of previous diseases and symptoms within 72 h after stroke onset. The community hospital physicians then reported confirmed stroke events, which were diagnosed using CT and/or magnetic resonance imaging (MRI), to TMUGH on a monthly basis. Suspected stroke events (i.e., those with stroke signs and symptoms but no confirmatory CT/MRI findings) were reported in real time and identified by TMUGH neurologists within 24 h.

### Definitions of Stoke Events and Outcomes

First-ever strokes were defined as the first recorded episode of rapidly developing neurological dysfunction (i.e., no history of stroke in the patient's medical records) caused by focal cerebral infarctions lasting >24 h (29). All stroke patients were those experiencing symptomatic strokes. Among these patients, none was diagnosed with a stroke during routine CT detection. Stroke events were categorized as either IS or intracerebral hemorrhage (ICH). Events were excluded if they involved transient ischemic attacks or subarachnoid hemorrhages.

**Figure 1:**
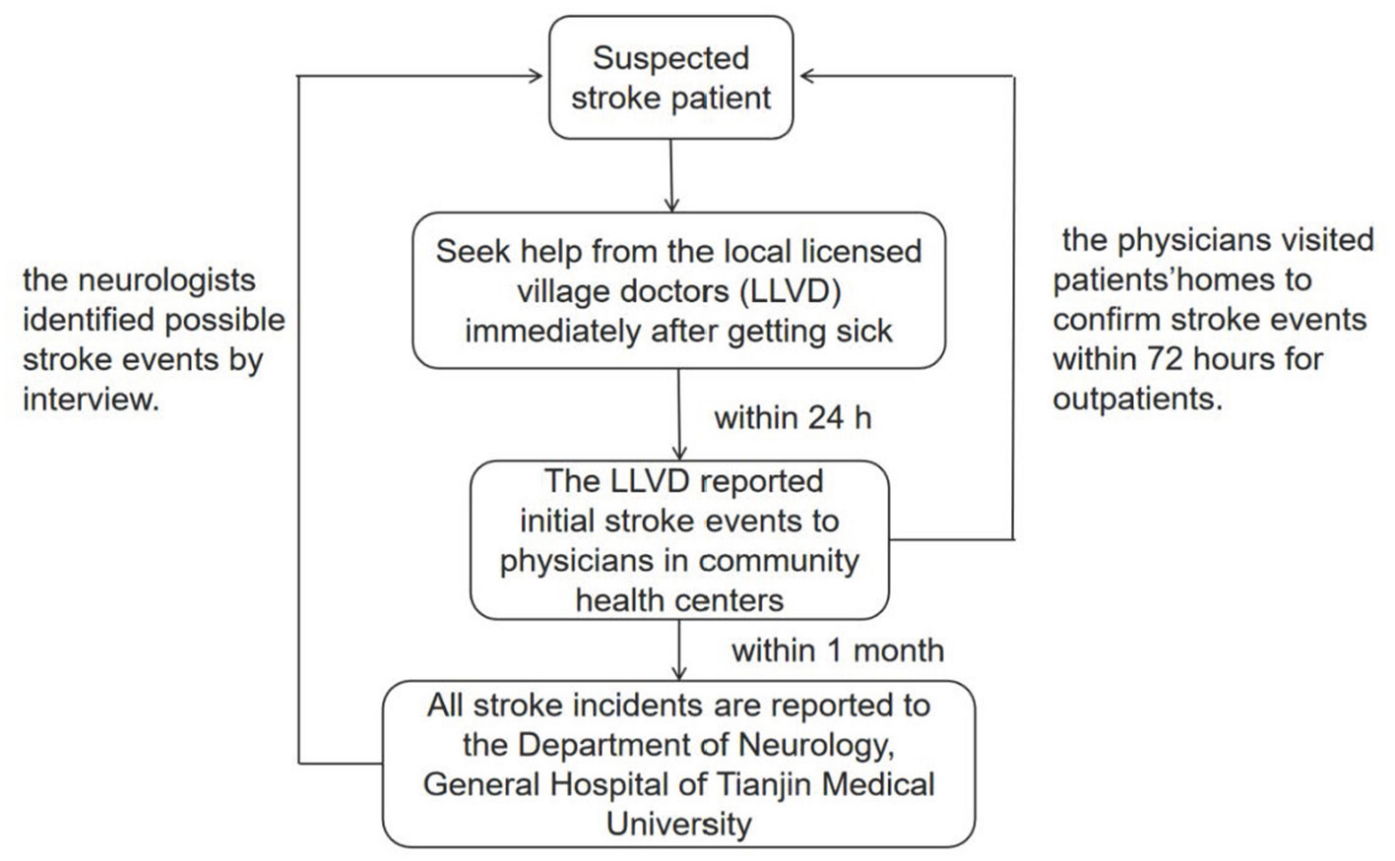
The three-level disease reporting system. This figure showed that the stroke of inpatients was diagnosed by the neurologist who was admitted to the hospital at that time; the patients who were not admitted were diagnosed according to the three-level report system, and finally by the neurologist of Tianjin Medical University General Hospital based on the patient's physical signs and imaging data. Finally, we will supplement the registration of stroke patients according to the local health system.

The stroke of inpatients was diagnosed by the neurologist who was admitted to the hospital at that time; the patients who were not admitted were diagnosed according to the three-level report system, and finally by the neurologist of Tianjin Medical University General Hospital based on the patient's physical signs and imaging data (Figure 1). Finally, we will supplement the registration of stroke patients according to the local health system.

Stroke management was evaluated based on the rates of neuroimaging diagnoses, hospitalizations, case fatalities, and recurrences. Case fatality was defined as death within 1 year after the initial stroke onset, excluding patients who died of non-stroke-related diseases. Recurrence was defined as a recurrent stroke within 1 year after the initial stroke event.

Stroke burden was assessed by the incidence of stroke and rates of case fatalities and recurrence among survivors within the first year after the initial stroke.

### Statistical Analysis

The demographic characteristics of women at the time of their first-ever stroke were summarized as means and standard deviations (SDs) for continuous variables and as numbers (percent) for categorical variables. Incidence rates per 100,000 person-years were calculated via direct standardization according to the 2010 World Health Organization population data (30). Incidence rates were calculated for neuroimaging diagnoses and hospitalizations during four study periods (1992–1998, 1999–2005, 2006–2012, and 2013–2019) with stratification according to stroke type and age group (<45 years, 45–64 years, and ≥65 years). The case fatality and 1-year recurrence rates were also calculated during the four study periods (1992–1998, 1999–2005, 2006–2012, and 2013–2018 years); variance analyses were performed to identify differences between the four periods. China implemented universal medical insurance at the end of 2008; therefore, this study also used 2008 as the cut-off point for analyzing changes in stroke incidence before and after the implementation of universal medical insurance.

Trends in stroke incidence were evaluated based on the annual percent change (APC), calculated as ln(rt) = a + bt; ln denotes the natural logarithm and t is the year. The trend (b) was estimated using ordinary regression and 100 × b represents the estimated APC in the stroke incidence. Negative APC values indicate a downward trend and positive values indicate an upward trend. All significance tests were two-sided, and differences were considered statistically significant at *p*-values of < 0.05. All statistical analyses were performed using SPSS software (version 25.0; IBM, Armonk, NY, USA).

## Results

### Demographic Characteristics During the Four Study Periods

A total of 597 low-income Chinese women experienced a stroke during the 193,385 person-years of surveillance. The mean age at stroke onset was 66.0 years (SD, 12.1 years), and there were no significant differences in the age at stroke onset among the four study periods. The mean education level clearly increased during the study period, with illiteracy rates decreasing from 74.2% during 1992–1998 to 34.2% during 2013–2019 (*P* < 0.001). Overall, 84.2% of the patients were married, 65.3% had official medical insurance, 88.4% had known histories of hypertension, 16.9% had known histories of diabetes, and 27.0% were disabled. Most of these proportions increased during the study period, with the exception of proportion having known histories of hypertension (all, *P* < 0.05; Table 1).

**Table 1:** Descriptive characteristics of women patients with first-ever stroke by period in the Tianjin Brain Study.

**Characteristic**	**Total**	**1992–1998**	**1999–2005**	**2006–2012**	**2013–2019**
Cases, *n* (%)	597	65	102	199	231
Person-year:	193,385	50,162	48,224	47,813	47,186
Age of onset, mean (SD), years	66.0 (12.1)	64.3 (11.7)	64.6 (12.6)	65.9 (12.5)	66.8 (11.7)
**Age groups**, ***n*** **(%)**					
<45 years	32 (5.4)	5 (7.6)	8 (7.9)	11 (5.5)	8 (3.5)
45–64 years	249 (41.7)	31 (47.0)	31 (30.7)	86 (43.2)	101 (43.7)
≥65 years	316 (52.9)	30 (45.5)	62 (61.4)	102 (51.3)	122 (52.8)
Education level: mean (SE), year:	2.8 (0.1)	0.9 (0.2)	1.5 (0.2)	2.8 (0.2)	3.8 (0.2)
**Educational groups**, ***n*** **(%)**					
0 year	285 (47.7)	49 (74.2)	66 (65.3)	91 (45.7)	79 (34.2)
1–6 years	244 (40.9)	15 (22.7)	30 (29.7)	93 (46.7)	106 (45.9)
>6 years	68 (11.4)	2 (3.0)	5 (5.0)	15 (7.5)	46 (19.9)
**Marital status**, ***n*** **(%)**					
Never married	1 (0.2)	0	0	0	1 (0.4)
Married	490 (84.2)	56(90.3)	91 (92.9)	163 (83.2)	180 (79.6)
Divorced	2 (0.3)	0	0	0	2 (0.9)
Widowed	89 (15.3)	6 (9.7)	7 (7.1)	33 (16.8)	43 (19.0)
**Medical insurance**, ***n*** **(%)**					
Official	390 (66.2)	11 (16.9)	31 (32.0)	166 (83.4)	182 (78.8)
Civil	199 (33.8)	51 (83.1)	66 (68.0)	33 (16.6)	49 (21.2)
**Hypertension**, ***n*** **(%)**					
Yes	528(88.4)	56 (86.2)	92 (90.2)	183 (92.0)	197 (85.3)
No	68 (11.4)	9 (13.8)	9 (8.8)	16 (8.0)	34 (14.7)
Unknown	1 (0.2)	0	1 (1.0)	0	0
**Diabetes**, ***n*** **(%)**					
Yes	101 (16.9)	3 (4.6)	9 (9.1)	32 (16.1)	57 (24.7)
No	446 (74.7)	62 (95.4)	90 (90.9)	164 (82.4)	130 (56.3)
Unknown	52 (8.7)	0	0	3 (1.5)	49 (19.0)
**Disability**, ***n*** **(%)**					
mRS ≥ 3	154 (27.0)	24 (36.9)	38 (37.3)	45 (22.6)	47 (22.9)
mRS <2	402 (70.4)	41 (63.1)	63 (61.7)	154 (77.4)	144 (70.3)
Missing	15 (2.6)	0	1 (1.0)	0	14 (6.8)

### Stroke Burden and Management During the Four Study Periods

**Figure 2:**
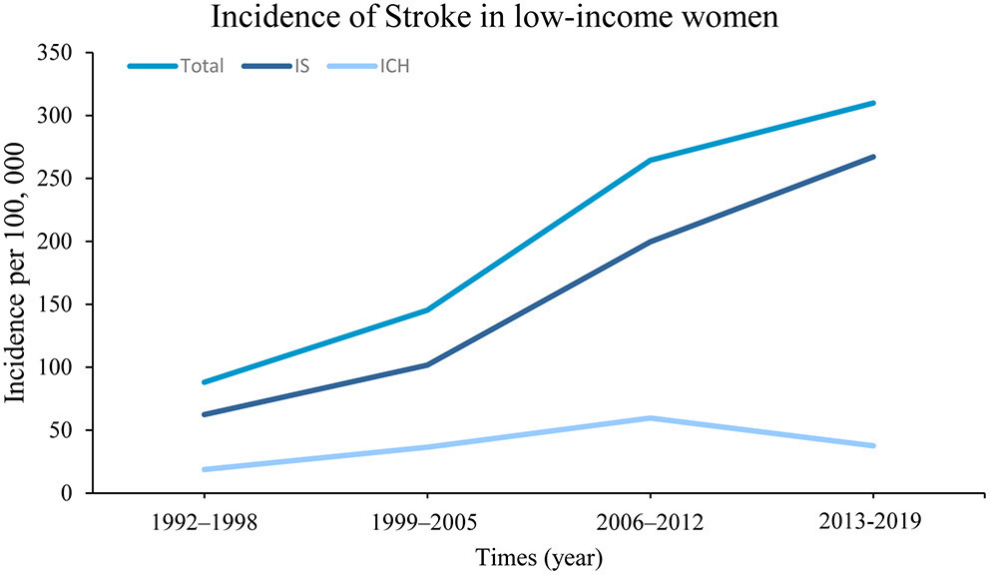
The annual percent change (APC) of stroke incidence from 1992 to 2019. This figure showed that the incidence of first-ever stroke increased by 6.4%, annually, between 1992 and 2019, with an APC of 7.8% for the incidence of IS (all, *P* < 0.001).

The incidence of first-ever stroke increased significantly during the study periods, with incidences per 100,000 person-years of 88.1 cases during 1992–1998, 145.4 during 1999–2004, 264.3 during 2005–2012, and 309.8 cases during 2013–2019 (*P* < 0.001). Similar trends were observed in the incidences of ICH (*P* = 0.027) and IS (*P* < 0.0.01). Moreover, the incidence of first-ever stroke increased by 6.4% (4.8, 8.3%, *P* < 0.001) annually overall, between 1992 and 2019, with an APC of 7.8% (6.0, 9.5%, *P* < 0.001) for IS (Figure 2).

Moreover, the incidence of overall stroke and hemorrhagic stroke between 2009 and 2019 was significantly lower than that between 1992 and 2008 (overall stroke, 307.0 per 100,000 person-years <479.3 per 100,000 person-years, *P* < 0.001; ICH, 241.2 per 100,000 person-years <397.2 per 100,000 person-years, *P* < 0.001) (Supplementary Table 1).

The rates of neuroimaging diagnoses and hospitalizations increased significantly during the four study periods and across all stroke types (all *P* < 0.05). The rate of neuroimaging diagnoses increased from 50.0% during 1992–1998 to 93.1% during 2013–2019, while the hospitalization rate increased from 3.0% during 1992–1998 to 58.9% during 2013–2019 (*P* < 0.001). The neuroimaging diagnosis and hospitalization rates increased significantly for both IS and ICH, whereas the recurrence rate decreased for all stroke types. The case fatality and 1-year recurrence rates decreased significantly during the study period for both overall stroke and IS (both, *P* < 0.001). However, the case fatality rate only decreased among patients with IS, but not among patients with ICH (Table 2).

**Table 2:** Stroke burden and management in low-income women by stroke types.

**Characteristic**	**1992–1998**	**1999–2005**	**2006–2012**	**2013–2019**	**APC for incidence (%)**
**Incidence of stroke, 1/100,000 (95%CI)**					
Total	88.1 (62.2, 114.1)	145.4 (111.7, 179.0)	264.3 (218.2, 310.3)	309.8 (259.7, 360.0)^*^	6.4 (4.7, 8.0)^*^
ICH	18.7 (−0.6, 37.9)	36.5 (19.6, 53.4)	59.6 (37.7, 81.4)	37.5 (20.1, 55.0)^*^	0.2 (−0.8, 1.2)
IS	62.4 (40.6, 84.3)	101.6 (73.5, 129.8)	199.7 (159.7, 239.7)	267 (220.4, 313.5)^*^	7.6 (5.7, 9.6)^*^
**Diagnosed by neuroimaging, %**					
Total	33 (50.0)	71 (70.3)	163 (81.9)	215 (93.1)^*^	—
ICH	10 (66.7)	24 (96.0)	43 (97.7)	25 (96.2)^*^	—
IS	23 (50.0)	47 (66.2)	120 (79.5)	190 (94.5)^*^	—
**Hospitalization rate, %**					
Total	2 (3.0)	14 (13.9)	66 (33.2)	136 (58.9)^*^	—
ICH	2 (13.3)	7 (28.0)	22 (50.0)	19 (73.1)^*^	—
IS	0 (0)	7 (9.9)	44 (29.1)	117 (58.2)^*^	—
**Case fatality, %**					
Total	58 (87.9)	79 (78.2)	101 (50.8)	43 (18.6)^*^	—
ICH	14 (93.3)	19 (76.0)	25 (56.8)	14 (53.8)	—
IS	39 (84.8)	55 (77.5)	72 (47.7)	25 (12.4)^*^	—
**Recurrence rate, %**					
Total	16 (24.2)	34 (33.7)	60 (30.2)	27 (11.7)^*^	—
ICH	4 (26.7)	5 (20.0)	15 (34.1)	2 (7.7)	—
IS	12 (26.7)	29 (40.8)	45 (29.8)	25 (12.4)^*^	—

*ICH, intracerebral hemorrhage; IS, ischemic stroke; SAH, subarachnoid hemorrhage*.

* *Presented P for trends < 0.05*.

### Age-Related Stroke Burden and Management

The incidence of first-ever stroke remained stable among patients who were <45 years old, although it increased markedly among patients who were ≥45 years old (*P* < 0.001). For example, stroke incidences increased 2.38-fold among patients who were 45–64 years old and 2.7-fold among patients who were ≥65 years old. The neuroimaging diagnosis and hospitalization rates also increased significantly among patients who were 45–64 years old and ≥65 years old. Among patients who were ≥65 years old, the neuroimaging diagnosis rate increased from 20.0% during 1992–1998 to 87.7% during 2013–2019. However, the case fatality rates within 1 year after stroke onset decreased from 83.9 to 5.9% among patients who were 45–64 years old and from 100 to 28.7% among patients who were ≥65 years old (both, *P* < 0.001). The recurrence rates also decreased significantly during the study period in all age groups (all, *P* < 0.05) (Table 3).

**Table 3:** Stroke burden and management in low-income women by age.

**Characteristic**	**1992–1998**	**1999–2005**	**2006–2012**	**2013–2019**	***P***
**Incidence of stroke, 1/100,000 (95%CI)**					
<45 years	18.3 (2.3, 34.3)	30.1 (9.3, 51)	43 (17.6, 68.5)	32.2 (9.9, 54.5)	0.247
45–64 years	177.7 (115.2, 240.2)	182.5 (118.3, 246.7)	516 (407.2, 624.8)	600.7 (483.9, 717.5)	<0.001
≥65 years	555.7 (357.4, 753.9)	1084.1 (815.7, 1352.5)	1,826 (1474.9, 2177.1)	2207.3 (1,820, 2594.7)	<0.001
**Diagnosed by neuroimaging**, ***n*** **(%)**					
<45 years	4 (80.0)	8 (100.0)	11 (100.0)	8 (100.0)	0.134
45–64 years	23 (74.2)	29 (93.5)	82 (95.3)	100 (99.0)	<0.001
≥65 years	6 (20.0)	34 (54.8)	70 (68.6)	107 (87.7)	<0.001
**Hospitalization rate**, ***n*** **(%)**					
<45 years	0 (0)	5 (62.5)	4 (36.4)	4 (50.0)	0.146
45–64 years	2 (6.5)	7 (22.6)	37 (43.0)	61 (60.4)	<0.001
≥65 years	0 (0)	2 (3.2)	25 (24.5)	71 (58.2)	<0.001
**Case fatality**, ***n*** **(%)**					
<45 years	2 (40.0)	3 (37.5)	2 (18.2)	2 (25.0)	0.735
45–64 years	26 (83.9)	21 (67.7)	21 (24.4)	6 (5.9)	<0.001
≥65 years	30 (100.0)	55 (88.7)	78 (76.5)	35 (28.7)	<0.001
**Recurrence rate**, ***n*** **(%)**					
<45 years	2 (40.0)	3 (37.5)	0 (0.0)	0 (0.0)	0.034
45–64 years	11 (36.7)	15 (48.4)	34 (39.5)	14 (13.9)	<0.001
≥65 years	3 (10.0)	16 (25.8)	26 (25.5)	13 (10.7)	0.007

## Discussion

To the best of our knowledge, this is the first population-based study regarding stroke burden and management among low-income Chinese women that has provided data regarding trends over a 28-year study period. The findings suggest that the burden of stroke remains severe in China, with a significant increase in the incidence of first-ever stroke across all stroke types among low-income women, and especially among women ≥45 years old. However, significant improvements were also observed in stroke management, and especially among women ≥45 years old. For example, significant increases were observed in the rates of neuroimaging diagnoses and hospitalizations, while decreases were observed in the IS and ICH recurrence rates along with a significant decrease in the IS case fatality rate.

There has been a decreasing trend in stroke incidence during recent decades in some developed countries (31), although the incidence of stroke among women has been increasing in most countries and especially in low-income countries (20, 32, 33). The incidence of stroke among Japanese women decreased from 123.1 cases per 100,000 person-years in 2008 to 97.0 cases per 100,000 person-years in 2017 (31). Despite the decreasing trend in stroke incidence, the incidence among women has remained relatively stable, with strokes affecting ~55,000 more women than men each year in the US (32). The Isfahan (Iran) Salt Study also revealed that the overall stroke incidence increased 6.65% per year between 2003 and 2009, and that the increase reached 11.5% per year among patients >50 years old (33). Our research also indicated that the burden of stroke has risen sharply in rural areas of China, with the burden among women increasing faster than that among men, based on annual incidence increases of 5.8% among men and 8.0% among women, between 1992 and 2012 (20). The present study also revealed an increasing burden of stroke among low-income Chinese women and especially among women ≥45 years old, with the overall incidence of stroke increasing from 88.1 cases per 100,000 person-years during 1992–1998 to 309.8 cases per 100,000 person-years during 2013–2019. In this context, economic development has some effects on the spectrum of diseases, with the stroke burden being particularly severe in low- and middle-income countries (19, 34). Thus, the increasing burden of stroke among Chinese women may be due to their relatively low socioeconomic status, especially in areas and population groups that already have low socioeconomic status (19, 34, 35). Moreover, previous studies have pointed out that the risk factors for stroke in this area are on the rise, especially obesity and fasting blood sugar increase, which is also an important reason for the increase in the incidence of stroke (23). In addition, China's healthcare system was reformed in 2009, which may have increased the hospital admission rate of stroke patients to a certain extent. The previous research results of the research team also showed that the prognosis of stroke patients in this area has improved significantly after the Health care reform (36). However, stroke events in this study do not only include hospitalized patients, but also all stroke events in the stroke surveillance system in the region. Therefore, the impact of medical reforms in this study on the incidence of stroke is very weak.

The Framingham study revealed that women had a 7% higher post-stroke case fatality rate and a 19% higher dependence rate than men (6). Further, another study indicated that the 1-month case fatality rate was higher among women than among men (13.9 vs. 12.1%) (37). Moreover, data from the Framingham study revealed that the 30-day post-stroke fatality rate among women did not change significantly over a 50-year period (from 21 to 20%) (38). The Brain Attack Surveillance in Corpus Christi Project also revealed that the rate of standard diagnostic testing was 2–9% lower among female stroke patients than among male stroke patients, in 2002, highlighting the need to increase the quality of stroke testing and care among women (39). In China, women, especially elderly women, have somewhat limited access to healthcare that is often related to their low socioeconomic status. Nevertheless, the present study revealed that the post-stroke case fatality rate decreased from 87.9% during 1992–1998 to 18.6% during 2013–2019. In addition, we observed significant increases in the neuroimaging diagnosis and hospitalization rates, which suggest that stroke management has significantly improved for low-income Chinese women. These changes may be related to implementation of the Chinese government's 2009 healthcare policy and the development of the Chinese economy. For example, before 2009, CT-based examinations for stroke were uncommon (40) and health insurance did not cover 79% of rural residents and 45% of urban residents. However, health insurance now covers >95% of Chinese citizens (41), and has increased access to healthcare and related resources for rural residents. We also observed that the hospitalization rate increased from 3.0% during 1992–1998 to 58.9% during 2013–2019, which may also be related to healthcare reform and increasing incomes in China that help provide better access to medical care (42). The increasing hospitalization rate and decreasing case fatality rate might also be attributed to improved medical imaging, greater knowledge of stroke symptoms among physicians and patients, and the creation of stroke wards across the country (43). The decrease in the stroke recurrence rate among low-income Chinese women might also be related to the improved effectiveness of secondary prevention efforts in rural areas.

The present study has several limitations. First, data were obtained from the TBS (20), which was conducted in a single township in northern China and does not provide nationally representative data. However, the study collected data regarding stroke incidence and management over a 28-year period, helping to clarify the trends in stroke burden for the studied population. Second, the study did not collect patient-level data regarding income, precluding an analysis of the relationship between personal income and stroke incidence. Nevertheless, women in the TBS were typically unemployed and predominantly derived their income from food production, with annual per capita incomes of < $100 USD in 1991 and < $2500 USD in 2018 (5). Thus, given the very low income levels, income stratification would have been unlikely to influence the incidence of stroke in the different income groups. Third, the effects of increases in education and income on medical treatment habits were not considered. Although the overall education level and income of the study population increased, previous studies showed that the secondary stroke prevention rate is still very low in the target population (44, 45) because the local residents' medical habits remained unchanged. Fourth, it is undeniable that changes in diet have a great influence on the risk of stroke. Due to limited conditions, this study did not collect data on the dietary structure of the population in the region from 1992 to 2019. The resulting results may exaggerate the role of health care reform. We will pay attention to supplementing relevant information in follow-up research. Finally, we did not collect data regarding stroke-related medications, although use of secondary prevention drugs is extremely uncommon in rural areas (44, 45).

## Conclusions

These findings suggested that further improvements are needed to manage stroke risk factors and improve healthcare coverage to reduce the stroke burden among low-income Chinese women, especially those who are middle-aged or older.

## Data Availability Statement

The raw data supporting the conclusions of this article will be made available by the authors, without undue reservation.

## Ethics Statement

The studies involving human participants were reviewed and approved by the Ethics Committee of Tianjin Medical University General Hospital. The patients/participants provided their written informed consent to participate in this study.

## Author Contributions

JW, XN, and YW were involved in conception and design and data interpretation for this article. XT, JL, CY, YH, CZ, QL, XinZ, DG, QY, XinyZ, and JT were involved in data collection, case diagnosis, and confirmation for this article. XT, JL, CY, and YH were involved in manuscript drafting. JW was involved in data analysis for this article. XN and JW were involved critical review in for this article. All authors contributed to the article and approved the submitted version.

## Funding

This study was supported partly by Key International (Regional) Cooperation and Exchange Projects of National Natural Science Foundation of China (71910107004).

## Conflict of Interest

The authors declare that the research was conducted in the absence of any commercial or financial relationships that could be construed as a potential conflict of interest.

## Publisher's Note

All claims expressed in this article are solely those of the authors and do not necessarily represent those of their affiliated organizations, or those of the publisher, the editors and the reviewers. Any product that may be evaluated in this article, or claim that may be made by its manufacturer, is not guaranteed or endorsed by the publisher.
