# Supplementary material for: Long-Term Trends in Stroke Management and Burden Among Low-Income Women in a Rural Area From China (1992–2019): A Prospective Population-Based Study
Source: Front Neurol. 2021 Oct 22;12:720962. doi: 10.3389/fneur.2021.720962 (PMC8569256; doi:10.3389/fneur.2021.720962)
Supplement: Supplementary file 1 [file Table_1.DOCX]

Supplemental Table 1. Stroke burden in low-income women by stroke types

| Characteristic | 1992-2019 | 1992-2008 | 2009-2019 | P |
| --- | --- | --- | --- | --- |
| Incidence of stroke, 1/100000 (95%CI): | | |  |  |
| Total | 200.5 (175.2, 225.8) | 479.3 (429.6, 529.0) | 307.0 (282.4, 331.6) | < 0.001 |
| IS | 144.8 (123.3, 166.3) | 397.2 (351.9, 442.4) | 241.2 (219.4, 263.0) | < 0.001 |
| ICH | 45.8 (33.7, 57.9) | 74 (54.5, 93.6) | 56.6 (46.0, 67.1) | 0.137 |
| Unknown | 10 (4.3, 15.6) | 8.1 (1.6, 14.5) | 9.3 (5.0, 13.5) | 0.801 |
